# Incidence Rate and Predictors of Intracranial Hemorrhage in Patients With Atrial Fibrillation: A Report From the Nationwide COOL‐AF Registry

**DOI:** 10.1002/clc.70040

**Published:** 2024-12-11

**Authors:** Rungroj Krittayaphong, Kasem Ratanasumawong, Komsing Methavigul, Chaiyasith Wongvipaporn, Gregory Y. H. Lip

**Affiliations:** ^1^ Faculty of Medicine Siriraj Hospital Mahidol University Bangkok Thailand; ^2^ Police General Hospital Bangkok Thailand; ^3^ Department of Cardiology Central Chest Institute of Thailand Nonthaburi Thailand; ^4^ Srinakarind Hospital, Faculty of Medicine Khon Kaen University Khon Kaen Thailand; ^5^ Liverpool Centre for Cardiovascular Science University of Liverpool and Liverpool Heart & Chest Hospital Liverpool UK

**Keywords:** atrial fibrillation, intracranial hemorrhage, oral anticoagulants, warfarin

## Abstract

**Background:**

Specific risk predictor scores of intracranial hemorrhage (ICH) risk in Asian subjects are lacking. We determined the incidence rate and predictors of ICH in patients with non‐valvular atrial fibrillation (AF).

**Methods:**

A prospective nationwide registry of patients with AF was conducted from 27 hospitals in Thailand. The adjudicated primary outcome was the development of ICH during follow‐up. Multivariable Cox proportional hazard model was performed to identify the independent predictors for ICH. A predictive model for ICH risk was developed and validated by bootstrap, calibration plot, C‐statistics, and decision curve analysis using our own data.

**Results:**

We studied a total of 3405 patients (mean age 67.8 years; 58.2% male) with an average follow‐up duration of 31.8 ± 8.7 months, during which ICH developed in 70 patients (2.06%). The incidence rate of ICH was 0.78 (0.61−0.98) per 100 person‐years. Predictors of ICH were chosen from the theory‐driven approaches in combination with the results of the univariable analysis. The predictive risk model had a c‐index of 0.717 (0.702−0.732) with good calibration, internal validation, and clinical usefulness using decision curve analysis. The probability of ICH at 3 years for an individual patient derived from the prediction model was compared with the probability derived from HAS‐BLED score by using the C‐statistics. The ICH probability from the COOL‐AF model was superior to the HAS‐BLED score in the prediction of ICH.

**Conclusion:**

The incidence rate of ICH was 0.78 (0.61−0.98) per 100 person‐years. Predictors of ICH were older age, male sex, nonsmoking, renal replacement therapy, and use of oral anticoagulants.

## Graphical abstract

The flow diagram of the COOL‐AF population, the incidence rate of intracranial hemorrhage (ICH), the ability to predict ICH from the model, and the ICH rate according to the oral anticoagulant strategy.

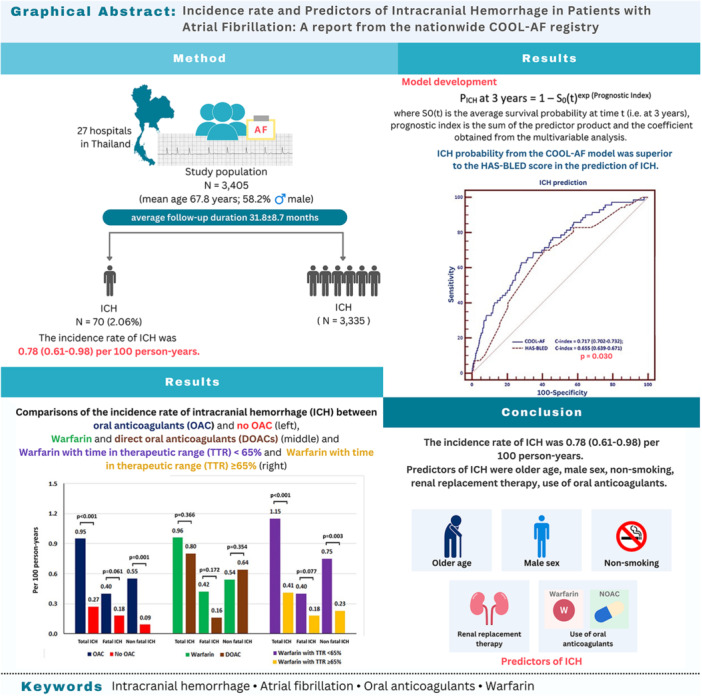

## 1 Introduction

Oral anticoagulant (OAC) is recommended for stroke prevention in patients with non‐valvular atrial fibrillation (AF), except in those with very low risk according to the CHA_2_DS_2_‐VASc score [1, 2]. The use of OAC increases the risk of major bleeding, including intracranial hemorrhage (ICH). The propensity to bleeding (including ICH) is also higher in Asian people compared to non‐Asians [3, 4, 5]. Major practice guidelines suggested the use of direct oral anticoagulants (DOAC) over warfarin as a Class I recommendation due to the lower incidence of ICH [1, 6, 7, 8]. However, warfarin remains the preferred OAC in many countries, especially in low or low‐middle‐income countries due to the reimbursement policy to limit the use of the high‐cost medications [9, 10, 11]. ICH prediction model has been reported from the GARFIELD‐AF and ORBIT‐AF registry [12]. However, the results from a registry may not be applied in other population since each population may have different rate predictors of ICH. The combined data set of the GARFIELD‐AF and ORBIT‐AF registry has the incidence rate of ICH of 0.31 per 100 person‐years whereas our AF population had the incidence rate of ICH 0.78 per 100 person‐years [13]. Prediction model usually derived from the variables from the multivariable Cox model and the validation of the prediction model was usually performed by bootstrap, calibration plot, and C‐statistics [14].

The objectives of this study were first, to determine the incidence rate of ICH in a nationwide AF registry; and second, to determine the predictors for ICH which would facilitate derivation and validation of a risk prediction model in Asians. Patients can be anticoagulated or non‐anticoagulated.

## 2 Methods

### 2.1 Study Population and Study Protocol

We enrolled patients with non‐valvular AF aged ≥ 18 years in a nationwide cohort, the COhort of antithrombotic use and Optimal INR Level in patients with non‐valvular AF in Thailand (COOL‐AF registry). Both anticoagulated and non‐anticoagulated patients can be enrolled in this study. The enrollment period was from 2014 to 2017. The inclusion and exclusion criteria were described in the previous report [9]. In brief, the exclusion criteria were patients with mechanical prosthetic valves, rheumatic valve disease, or AF from transient reversible causes were excluded from the registry. The study protocol was approved by the Central Research Ethics Committee (CREC) with the Certificate of Approval (COA) number CREC 003/2014. Written informed consent was obtained from all included patients before participation, and the study was conducted in accordance with the principles set forth in the Declaration of Helsinki and the International Conference on Harmonization for Good Clinical Practice Guidelines.

### 2.2 Data Collection

Data collection at baseline visit included demographic data, weight and height, vital sign, medical history, comorbid conditions, details of AF, symptoms and signs, investigation including ECG and echocardiogram, laboratory data including international normalized ratio (INR) for those who use warfarin, components of CHA_2_DS_2_‐VASc and HAS‐BLED score, medication data including OAC and antiplatelets. Data during follow‐up visits were collected in a similar manner every 6 months. Among patients with warfarin, time in the therapeutic range (TTR) was calculated by the Rosendaal method [15]. The target range of INR for the TTR calculation was 2.0−3.0 as recommended in the 2020 ESC guideline for the management of AF [6]. In summary, we collected INR data and the date of the INR test of every visit. From the two adjacent INR results, we estimated the percentage of time between the two adjacent visits that have the INR within the range of 2−3. The days within the range of the two INR intervals were calculated from the percentage of time within the range divided by the number of days between the two INR tests. We then calculated a sum of days within the range of every interval between the two INR tests. TTR is derived from the overall days within the range divided by the total number of days starting from the first INR test to the last INR test.

### 2.3 Outcomes

The main outcome of this study was ICH. Supporting documents were required to upload into the web‐system to support the occurrence of outcomes. The central data management team verified the data in the web system and the supporting document. The adjudication committee made the final conclusion for the confirmation of an event. ICH was defined as the bleeding within the skull which could be intracerebral bleeds, subarachnoid bleeds, subdural bleeds, and epidural bleeds. Each patient may have more than one type of ICH. According to the Bleeding Academic Research Consortium (BARC), ICH does not include microbleeds and secondary hemorrhagic transformation [16]. Secondary hemorrhagic transformation was excluded from the clinical and imaging information, that is, evidence of mottled hemorrhage within the larger area of brain infarction. ICH was classified as traumatic or non‐traumatic. The ICH event was considered traumatic when trauma was clearly stated in the medical record and the ICH was considered to be related to the trauma episode. Among those who had available imaging data (computerized tomography or magnetic resonance imaging), ICH volume was calculated by the ABC/2 formula [17]. The ICH volume calculation formula is A × B × C/2 where A is the greatest hemorrhage diameter in the axial plane, B is the hemorrhage diameter perpendicular to A in the axial plane, and C is the the number of CT slices with hemorrhage multiplied by the slice thickness. If the measurements are made in centimeters, the volume will be in cubic centimeters (cm^3^) or milliliters (mL) [18]. Fatal ICH was defined as death occurring during hospital admission due to ICH.

### 2.4 Statistical Analysis

Data were described as mean and standard deviation (SD) for continuous data, and count and percentages for categorical data. Comparisons of continuous data were made by the Student *t*‐test for independent data. For categorical data, the chi‐square test or Fisher exact test were used as appropriate. Incidence rates of all ICH, fatal, and nonfatal ICH, were reported as a rate per 100 person‐years.

The Cox proportional hazard model was used for univariable and multivariable analysis to determine factors that predict ICH. The selection of variables in the multivariable model was based on the prior knowledge of the covariates that might have an impact on the outcome and theory‐driven approaches that might be the predictors of each clinical outcome in combination with the results of the univariate analysis. Variables with *p* < 0.10 in the univariable analysis were selected for multivariable analysis using backward elimination with *p* < 0.05 as the stopping criteria. Sex was also included. The results of the univariable and multivariable analyses are presented as hazard ratios and adjusted hazard ratios, respectively, along with their 95% confidence intervals. The adjusted variables in the multivariable model were the following: age, AF type, symptomatic AF, hypertension, diabetes mellitus, smoking, hypercholesterolemia, renal replacement therapy (RRT), CKD, history of bleeding, antiplatelet, and anticoagulant.

### 2.5 Model Development

The 3‐year ICH risk prediction model was developed from the variables in Cox proportional hazard model of the multivariable analysis. The probability of ICH at 3 years can be calculated from the model formula:

$$P_{\text{ICH}}{\text{at 3 years}\;=1–S}_{0}{\left(t\right)}^{\text{exp}\;(\text{PrognosticIndex})}$$

where S_0_(t) is the average survival probability at time t (i.e., at 3 years), prognostic index is the sum of the predictor product and the coefficient obtained from the multivariable analysis.

### 2.6 Model Validation

The model was validated by bootstrap, calibration plot, C‐statistics, and decision curve analysis. Prediction model applicability and bias was assessed according to the recommendation from the Prediction model Risk Of Bias ASsessment Tool (PROBAST) [19]. Bootstrapping of 100 samples was performed to verify the fitted model. A calibration plot and calibration slope were developed to assess the agreement between the observed and predicted risk of ICH. The Harrel C‐statistics were used to measure model discrimination. The D‐statistics assess the discrimination ability of the fitted model into low‐risk and high‐risk patients [20]. C‐ and D‐statistics were also calculated after the 100 sample bootstraps as an internal validation. Apparent and optimism‐corrected values of the C‐ and D‐statistics were reported. The Cox proportional Hazard model was also used to test the differences in cumulative event rates between patients in different risk groups according to the predictive model. Brier score was evaluated to assess the predictive ability of the model [21]. The lower the Brier score, the more accurate the prediction. The ICH prediction ability of the developed model was tested with the predictive model of the HAS‐BLED score. The DeLong test [22] was used to compare the area under the receiver‐operating‐characteristics (ROC) curve, that is, C‐statistics, between two models. Both model development and validation was performed by using only the data set of the COOL‐AF registry with guidance of the Transparent Reporting of a multivariable prediction model for Individual Prognosis or Diagnosis (TRIPOD) reporting guideline [14].

A restricted cubic spline graph was performed for the assessment of the relation of TTR and ICH among those who were on warfarin. TTR was treated as continuous data on the *X*‐axis and the hazard ratio and 95% confidence interval was displayed on the *Y*‐axis.

Sensitivity analysis was performed for the assessment of predictive model for ICH only amongst patients who were on OAC. All statistics were performed by the SPSS statistical software version 18.0 (SPSS Inc., Chicago, IL, USA) and R version 3.6.3 (www.r-project.org).

## 3 Results

**Figure 1:**
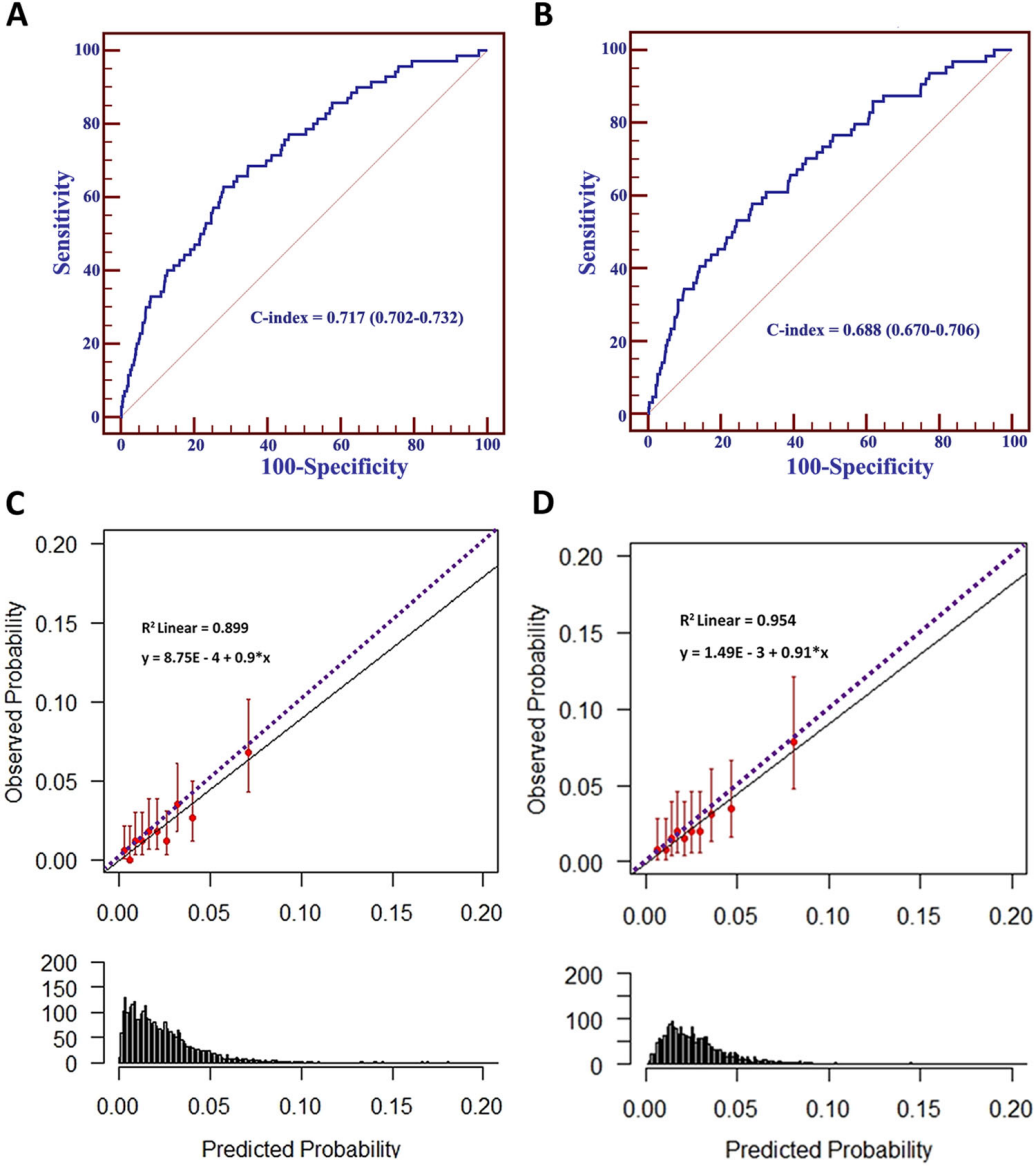
(A, B) Receiver operating characteristic graph of a predictive model for intracranial hemorrhage (ICH) for all patients (A) and patients with oral anticoagulants (B). (C, D) Calibration plots show a good agreement of the probability of predicated and observed ICH for all patients (C) and patients with oral anticoagulants (D).

We studied a total of 3405 patients (mean age 67.8 ± 11.3 years; 58.2% male). Supporting Information S1: Figure 1 shows the flow of study population. Table 1 shows baseline characteristics of patients with and without ICH. Mean TTR was slightly lower in patients with ICH compared to no ICH group (46.7 ± 23.2% vs. 53.7 ± 26.4%, *p* = 0.057). The proportion of patients with TTR < 65% was greater in patients with ICH compared to those without ICH (82.7% vs. 63.7%, *p* = 0.005). Baseline INR was not different between ICH and no ICH groups (2.2 ± 0.7 vs. 2.1 ± 0.9, *p* = 0.377).

**Table 1:** Baseline characteristics of study population with and without intracranial hemorrhage (ICH).

Variables	All (*N* = 3405)	ICH (*N* = 70)	No ICH (*N* = 3335)	*p* value
Age (years)	67.8 ± 11.3	73.0 ± 8.5	67.7 ± 11.3	***< 0.001***
Female gender	1424 (41.8%)	26 (37.1%)	1398 (41.9%)	0.423
Time after diagnosis of AF (years)	3.4 ± 4.3	3.3 ± 4.0	3.4 ± 4.3	0.925
Atrial fibrillation				0.231
‐ Paroxysmal	1148 (33.7%)	18 (25.7%)	1130 (33.9%)	
‐ Persistent	645 (18.9%)	12 (17.1%)	633 (19.0%)	
‐ Permanent	1612 (47.3%)	40 (57.1%)	1572 (47.1%)	
Symptomatic AF	2620 (76.9%)	48 (68.6%)	2572 (77.1%)	0.093
History of heart failure	913 (26.8%)	16 (22.9%)	897 (26.9%)	0.450
History of coronary artery disease	547 (16.1%)	10 (14.3%)	537 (16.1%)	0.682
Cardiac implantable electronic device	341 (10.0%)	6 (8.6%)	335 (10.0%)	0.684
History of ischemic stroke/TIA	592 (17.4%)	13 (18.6%)	579 (17.4%)	0.791
Hypertension	2330 (68.4%)	55 (78.6%)	2275 (68.2%)	0.065
Diabetes mellitus	839 (24.6%)	24 (34.3%)	815 (24.4%)	0.058
Smoking	678 (19.9%)	7 (10.0%)	671 (20.1%)	***0.036***
Hypercholesterolemia	1917 (56.3%)	48 (68.6%)	1869 (56.0%)	***0.036***
Renal replacement therapy	40 (1.2%)	4 (5.7%)	36 (1.1%)	***0.009***
Dementia	29 (0.9%)	1 (1.4%)	28 (0.8%)	0.454
CKD	1756 (51.6%)	50 (71.4%)	1706 (51.2%)	***0.001***
History of bleeding	324 (9.5%)	5 (7.1%)	319 (9.6%)	0.494
CHA_2_DS_2_‐VASc score				**0.007**
‐ Low	287 (8.4%)	2 (2.9%)	285 (8.5%)	
‐ Intermediate	548 (16.1%)	4 (5.7%)	544 (16.3%)	
‐ High	2570 (75.5%)	64 (91.4%)	2506 (75.1%)	
HAS‐BLED score				0.517
‐ 0	490 (14.4%)	7 (10.0%)	483 (14.5%)	
‐ 1−2	2375 (69.7%)	50 (71.4%)	2325 (69.7%)	
‐ ≥ 3	540 (15.9%)	13 (18.6%)	527 (15.8%)	
Baseline INR	2.1 ± 0.9	2.2 ± 0.7	2.1 ± 0.9	0.377
TTR (%)	53.6 ± 26.4	46.7 ± 23.2	53.7 ± 26.4	0.057
TTR < 65%	1432 (64.1%)	43 (82.7%)	1389 (63.7%)	***0.005***
Antiplatelet	892 (26.2%)	13 (18.6%)	879 (26.4%)	0.143
Anticoagulant	2568 (75.4%)	64 (91.4%)	2504 (75.1%)	***0.002***
‐ Warfarin	2340 (68.7%)	59 (84.3%)	2281 (68.4%)	***0.005***
‐ DOACs	228 (6.7%)	5 (7.1%)	223 (6.7%)	0.808
Beta‐blocker	2477 (72.7%)	46 (65.7%)	2431 (72.9%)	0.182
CCB	935 (27.5%)	27 (38.6%)	908 (27.2%)	***0.035***
Digitalis	539 (15.8%)	11 (15.7%)	528 (15.8%)	0.979
MRA	280 (8.2%)	2 (2.9%)	278 (8.3%)	0.099
Statin	2014 (59.1%)	49 (70.0%)	1965 (58.9%)	0.062
ACEI/ARB	1557 (45.7%)	34 (48.6%)	1523 (45.7%)	0.629

*Note:* Bold and italic indicates significant *p*‐value.

Abbreviations: ACEI = angiotensin converting enzyme inhibitor, AF = atrial fibrillation, ARB = angiotensin receptor blocker, CCB = calcium channel blocker, CKD = chronic kidney disease, DOAC = direct oral anticoagulants, ICH = intracranial hemorrhage, MRA = mineralocorticoid receptor antagonist, TIA = transient ischemic attack.

### 3.1 ICH Outcomes

The average follow‐up duration was 31.8 ± 8.7 months, during which ICH developed follow‐up in 70 patients (2.06%). The incidence rate of ICH was 0.78 (0.61−0.98) per 100 person‐years. ICH resulted in death in 31 patients (44.3%). The incidence rates of fatal and nonfatal ICH were 0.34 (0.23−0.49) and 0.43 (0.31−0.59) per 100 person‐years, respectively. Types of ICH were demonstrated in 54 patients, while the type of ICH was not available in 16 cases (22.9%). Among 17 patients with intracerebral hemorrhage and available imaging data, one patient used no OAC and intracerebral hemorrhage volume was 28.5 mL, one patient use DOAC and the volume was 6.6 mL, and 15 patients used warfarin and the volume was 57.2 ± 59.3 mL. The details of ICH types are shown in Supporting Information S1: Table 1.

### 3.2 Univariable and Multivariable Analysis

Table 2 shows results of univariable and multivariable analysis. Independent predictors for ICH from multivariable model were older age, RRT, and OAC use. Protecting factors were female sex and smoking.

**Table 2:** Univariable and multivariable analysis for predictors of intracranial hemorrhage.

Variables	Univariable analysis		Multivariable analysis	
	HR (95% CI)	*p* value	HR (95% CI)	*p* value
Age (years)	1.05 (1.03−1.08)	***< 0.001***	1.05 (1.03−1.08)	***< 0.001***
Female sex	0.82 (0.51−1.34)	0.428	0.57 (0.35−0.94)	***0.027***
Atrial fibrillation				
‐ Paroxysmal	Reference	0.161		
‐ Persistent	1.23 (0.60−2.55)	0.580		
‐ Permanent	1.69 (0.97−2.95)	0.065		
Symptomatic AF	0.66 (0.40−1.09)	0.104		
History of heart failure	0.82 (0.47−1.43)	0.481		
History of coronary artery disease	0.89 (0.46−1.74)	0.730		
Cardiac implantable electronic device	0.82 (0.36−1.90)	0.651		
History of ischemic stroke/TIA	1.13 (0.62−2.06)	0.697		
Hypertension	1.69 (0.96−3.00)	0.071		
Diabetes mellitus	1.59 (0.97−2.60)	0.067		
Smoking	0.43 (0.20−0.95)	***0.036***	0.36 (0.16−0.81)	***0.013***
Dyslipidemia	1.66 (0.99−2.74)	0.050		
Renal replacement therapy	6.02 (2.19−16.52)	***< 0.001***	8.54 (3.08−23.67)	***< 0.001***
Dementia	1.82 (0.25−13.11)	0.552		
CKD	2.50 (1.49−4.20)	***0.001***		
History of bleeding	0.75 (0.30−1.86)	0.532		
Antiplatelet	0.64 (0.35−1.18)	0.151		
Anticoagulant	3.52 (1.52−8.12)	***0.003***	3.19 (1.37−7.42)	***0.007***
‐ Warfarin	2.49 (1.31−4.75)	***0.005***		
‐ DOACs	1.03 (0.42−2.56)	0.949		

*Note:* Bold and italic indicates significant *p*‐value.

Abbreviations: AF = atrial fibrillation, CI = confidence interval, CKD = chronic kidney disease, DOAC = direct oral anticoagulants, HR = hazard ratio, TIA = transient ischemic attack.

### 3.3 Model Development

The ICH prediction model of our study is P_ICH at 3 years _= 1 – 0.99965861^exp (Prognostic Index)^

where Prognostic index = 0.050544 × age (years) − 0.564094 × female gender − 1.018869 × smoking + 2.144524 × renal replacement therapy + 1.160677 × anticoagulant

### 3.4 Model Validation

**Figure 2:**
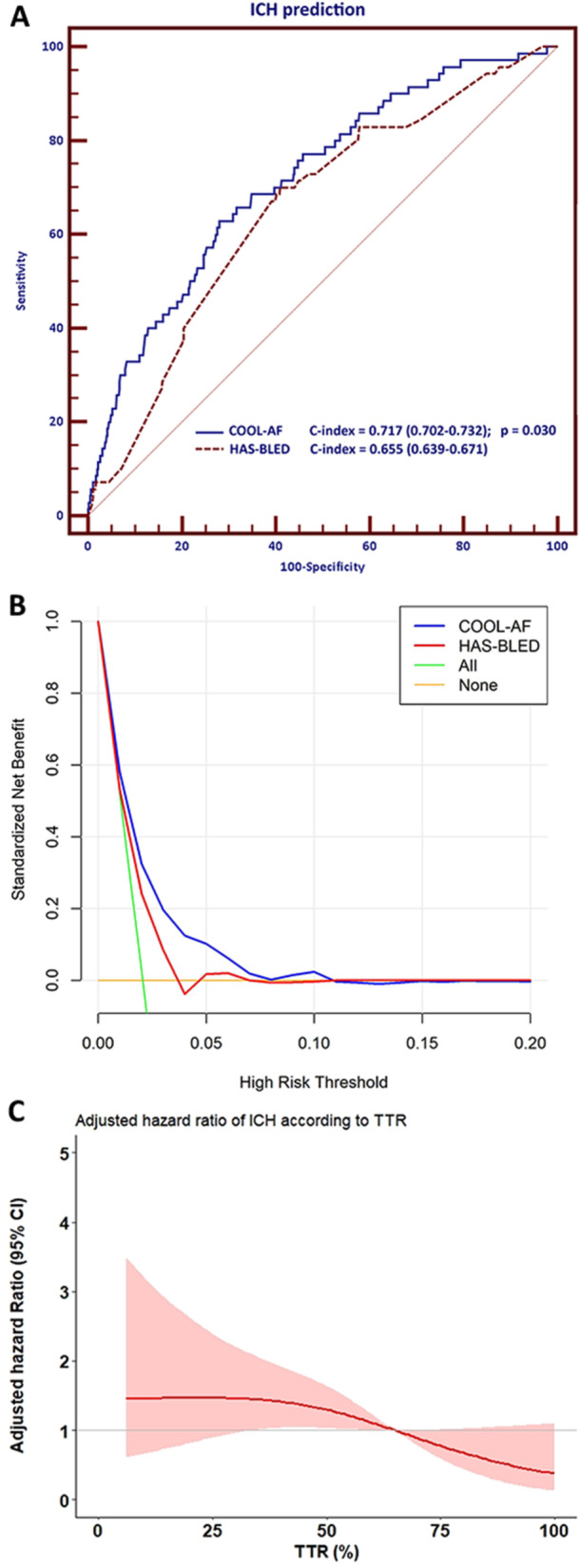
(A) Receiver‐operating‐characteristics graph of COOL‐AF model and HAS‐BLED model for the prediction of ICH. (B) Decision curve analysis also demonstrated the superiority of the COOL‐AF model as compared to the HAS‐BLED model. (C) Cubic spline graph of time in therapeutic range (TTR) on the *X*‐axis and hazard ratio for intracranial hemorrhage (ICH) on the *Y*‐axis. CI = confidence interval.

ROC graph displays the predictive ability of the model for ICH and the C‐statistic for the prediction of ICH at 3 years was 0.717 (0.702−0.732) for all patients and 0.688 (0.670−0.706) for patients with OAC (Figure 1A,B). We tested how well the predictive probability of the model can separate the cumulative ICH event rate by categorizing patients into five risk groups based on quintiles (1 = very low risk, 2 = low risk, 3 = intermediate risk, 4 = high risk, and 5 = very high risk). The plot of the hazard graph demonstrates a good separation of the risk of ICH among the five risk groups (Supporting Information S1: Figure 2, 1 = very low risk, 5 = very high risk). D‐statistic was 1.360 (0.974−1.746) which indicates a good discrimination of the model. The hazard ratio for ICH in the high‐risk group compared to the low‐risk group was 17.6 (4.2−73.6).

The C‐ and D‐statistics after the bootstraps was 0.715 (0.681−0.749) and 1.322 (1.104−1.540). The Brier score was 0.0199 which indicated that the model can nicely predict ICH events.

Calibration plots among 10 groups of patients showed good agreement between predicted probability derived from the prediction model and actual ICH outcomes with a calibration slope of 0.90, with an intercept of 0.0009 and *R* ^2^ of 0.899 (Figure 1C). Calibration slope and *R* ^2^ was slightly higher in patients with OAC (Figure 1D). After the correction of optimism, the C‐statistic, slope, and intercept of the calibration plot were 0.747 (0.745−0.750), 0.836, and −0.100, respectively.

The optimisms for the C‐statistics, slope, and intercept of the calibration plot were calculated using the bootstrap method. The optimisms were 0.027, 0.083, and 0.337 for the C‐statistics, slope, and intercept, respectively. After correcting the optimism, the C‐statistics, slope, and intercept of the calibration plot were 0.704 (0.689–0.719), 0.817, and 0.337, respectively.

### 3.5 Comparison With HAS‐BLED Score

The probability of ICH at 3 years for an individual patient derived from the prediction model was compared with the probability derived from the HAS‐BLED score by using the C‐statistics. The ICH probability from the COOL‐AF model was superior to the HAS‐BLED score in the prediction of ICH. We used all variables in the HAS‐BLED to develop the prediction model to avoid the limitation of the integers. The C‐index of COOL‐AF model was significantly greater than HAS‐BLED model (0.717 [0.702−0.732] vs. 0.655 [0.639−0.671]) (DeLong test *p *= 0.030) (Figure 2A). Decision curve analysis also demonstrated the superiority of the COOL‐AF ICH prediction model as compared to HAS‐BLED model (Figure 2B).

### 3.6 ICH and TTR Amongst Warfarin Users

Among patients with warfarin and ICH in our study, the median and interquartile range of INR during ICH was 2.62 (1.87, 4.03). From the restricted cubic spline graph of TTR on the *X*‐axis, and hazard ratio and 95% confidence interval of the ICH on the *X*‐axis, the risk of ICH increased when TTR decreased and the graph crossed the Hazard ratio 1.0 line at TTR 65% (Figure 2C). Among patients who were on warfarin, the unadjusted and adjusted hazard ratios and 95% confidence intervals of poor TTR control defined as TTR < 65% in the prediction of ICH were 3.12 (1.53−6.36), *p* = 0.002, and 3.09 (1.52−6.29), *p* = 0.002, respectively.

Supporting Information S1: Figure 3 compares the incidence rate of ICH between OAC and no OAC (left), warfarin in DOACs (middle), and warfarin with TTR < and ≥ 65% (right). This shows the higher rate of ICH on OAC and warfarin (vs. DOAC), with more fatal ICHs on warfarin compared to DOAC and amongst warfarin patients, if TTR was < 65%.

### 3.7 Sensitivity Analysis

Sensitivity analysis was performed for the assessment of predictive model for ICH only amongst patients who were on OAC (75.4% of the study cohort). Univariable and multivariable analysis for factors predicting ICH in patients who received OAC are shown in Supporting Information S1: Table 2. C‐statistics before and after the bootstrap were 0.688 (0.621−0.755) and 0.702 (0.669−0.736) respectively. The Brier score was 0.0262.

## 4 Discussion

In this multicenter nationwide AF registry, we demonstrate that among 3405 patients with average follow‐up duration of 31 months, the incidence rate of ICH was 0.78 per 100 person‐years and independent predictors for ICH were increased age, male gender, RRT, being on OAC and nonsmoking. Second, we derived a risk predictive model for ICH which had a C‐statistic of 0.717 (0.702−0.732) with good calibration, internal validation, and clinical usefulness using decision curve analysis, as well as superiority to the HAS‐BLED score. Third, amongst warfarin users, we show the optimal TTR was ≥ 65%, and the risk of ICH increased when TTR decreased.

The overall incidence rate of ICH in our study was 0.78 (0.61−0.98), while in patients who used and did not use OAC, the rates were 0.95 (0.73−1.21) and 0.27 (0.10−0.59), respectively. The incidence rate was comparable or higher than previous studies [10, 23, 24]. For example, the incidence rate of ICH in the four DOAC trials were 0.7−0.85 per 100 person‐years for the warfarin arm and 0.2−0.5 per 100 person years for DOAC users [23]. The FUSHIMI‐AF registry enrolled 3878 Japanese AF patients from community settings during 2011−2012. Since the FUSHIMI‐AF study is a registry in nature and from the Asian population, it shares many similarities with our study. They reported the incidence rate of ICH of 0.53 and 0.85 per 100 person‐years in females and males, respectively [24]. The higher ICH incidence rate in our study may be due to a higher proportion of warfarin in our study, and Asians tend to have a higher ICH rate compared to non‐Asians, especially when they are on OAC, particularly warfarin [5]. In the major DOAC trials, Asians had a risk of ICH two to three times compared to non‐Asians [3].

There are many predictors for ICH that have been reported from previous reports which were mainly from clinical trials of DOACs, such as RELY AF [25], ROCKET AF [26], and the ARISTOTLE study [27] (Table 3). These trials were performed in a clinical trial setting in AF patients with risk factors for stroke during 2005−2010. The number of study patients were 18 113, 14 264, and 18 201, respectively. Compared to our study, these trials are more control in term of treatment and follow‐up due to the clinical trial nature. Therefore, interpretation of the result comparison with our study have to take the differences into consideration. These predictors were age [25, 26, 27], previous stroke/TIA [25, 26, 27], warfarin use [25, 26, 27], aspirin use [25, 27], and Asian population [26, 27]. These data may not be perfectly applicable to the Asian population since the models were developed from multinational clinical studies with limited proportions of Asians.

**Table 3:** Summary of incidence rate and predictors of intracranial hemorrhage of published data that reported both incidence rate and predictors.

Variables	COOL‐AF	RELY AF	ROCKET AF	ARISTOTLE
Year of enrollment	2014−2017	2005−2007	2006−2009	2006−2010
Number of patients	3405	18 113	14 264	18 201
Country	Thailand	Multi‐national	Multi‐national	Multi‐national
Asian	100	2782 (15.4%)	932 (6.5%)	1993 (10.9%)
Age (mean or median)	68	71	73	70
Female (%)	41.8	36.4	39.7	35.2
Incidence rate of ICH (per 100 person‐years)				
‐Warfarin	0.96	0.76	0.7	0.80
‐DOACs	0.80	0.31 (dose 150) 0.23 (dose 110)	0.5	0.33
Predictors for ICH				
‐Age	√	√	√	√
‐Male	√			
‐Warfarin	√	√	√	√
‐Aspirin		√		√
‐Previous stroke/TIA		√	√	√
‐Asians	NA		√	√
‐Renal replacement therapy	√			
‐Nonsmoking	√			
‐Low serum albumin			√	
‐Reduced platelet count			√	
‐Increased DBP			√	
‐No history of HF			√	

Abbreviations: AF = atrial fibrillation, DBP = diastolic blood pressure, DOAC = direct oral anticoagulants, HF = heart failure, ICH = intracranial hemorrhage, TIA = transient ischemic attack.

Regarding INR during ICH in patients who were on warfarin, the results of the ARISTOTLE study demonstrated that 78% had INR less than 3.0 [27]. In our study, among patients with warfarin and ICH, median INR during ICH was 2.62 which means that majority of patients who had ICH during being on warfarin had INR less than 3 or within the target INR. We also show that the optimal TTR in warfarin users was ≥ 65%, as below this, the risk of ICH increased when TTR decreased. This result may indicate that the low TTR group may be the patients with labile INR which is associated with the increased risk of ICH which has been shown in the previous study [28].

The result of our study showed that smoking is a protective factor for ICH. We don't know if this is real or by chance. Some previous studies have demonstrated the smoker's paradox in patients with acute myocardial infarction. They demonstrated a better outcome in smokers compared to nonsmokers in terms of mortality and bleeding complications [29, 30]. A systematic review and meta‐analysis demonstrated that smoking has an interaction with warfarin by increasing warfarin clearance and reducing the warfarin effect which may be associated with a reduction in bleeding complications from warfarin [31].

### 4.1 Limitations

There were some limitations of this study. First, despite the fact that this study was a nationwide cohort study with prospective data collection, the imaging data of ICH were not obtained in all patients. However, the adjudication committee considered the supporting documents and confirmed the ICH events. Second, the majority of OAC use in our study was warfarin. Therefore, the results of our study may not be applied to many populations that have different patterns of OAC use. The risk model reported and validated is applicable to Asian patients with AF from Thailand, and ongoing analyses will validate the risk model in Asian patients from other Asian groups and assess its applicability to non‐Asians. Third, comparing the prediction model with HAS‐BLED may not be entirely fair, given that HAS‐BLED transforms regression coefficients into integers for simplicity, potentially impacting model performance.

## 5 Conclusion

The incidence rate of ICH was 0.78 (0.61−0.98) per 100 person‐years. Predictors of ICH were older age, male sex, nonsmoking, RRT, and use of oral anticoagulants. Using predictors of ICH, we derived and validated a new risk predictive model for ICH with good calibration, internal validation, and clinical usefulness.

## Author Contributions

All authors made substantial contributions to the conception and design, acquisition of data, or analysis and interpretation of data; took part in drafting the article or revising it critically for important intellectual content; agreed to submit to the current journal; gave final approval of the version to be published; and agree to be accountable for all aspects of the work.

## Ethics Statement

The study protocol was approved by Central Research Ethics Committee (CREC) with the Certificate of Approval (COA) number CREC 003/2014.

## Consent

Written informed consent was obtained from all included patients before participation, and the study was conducted in accordance with the principles set forth in the Declaration of Helsinki and the International Conference on Harmonization for Good Clinical Practice Guidelines.

## Conflicts of Interest

Gregory Y. H. Lip: Consultant and speaker for BMS/Pfizer, Boehringer Ingelheim, and Daiichi‐Sankyo. No fees are directly received personally. The other authors declare no conflicts of interest.

## Supporting information

Supporting information.

## Data Availability

All relevant data are within the manuscript.
