# Supplementary material for: Incidence Rate and Predictors of Intracranial Hemorrhage in Patients With Atrial Fibrillation: A Report From the Nationwide COOL‐AF Registry
Source: Clin Cardiol. 2024 Dec 11;47(12):e70040. doi: 10.1002/clc.70040 (PMC11635119; doi:10.1002/clc.70040)

**Supplementary data**

**Supplementary Table 1.** Types of intracranial hemorrhage (ICH)

| Types of ICH | N (%) | | | Overall N (%) | | |
| --- | --- | --- | --- | --- | --- | --- |
|  | All | Traumatic  (n = 12) | Non-traumatic  (n = 42) | All | Traumatic  (n = 12) | Non-traumatic  (n = 42) |
| Intracerebral | 21 (38.9%) | 2 (16.7%) | 19 (45.2%) | 28 (40.0%) | 3 (25.0%) | 25 (59.5%) |
| Subdural | 22 (40.7%) | 8 (66.7%) | 14 (33.3%) | 26 (37.1%) | 9 (75.0%) | 17 (40.5%) |
| Subarachnoid | 4 (7.4%) | 1 (8.3%) | 3 (7.1%) | 8 (11.4%) | 1 (8.3%) | 7 (16.7%) |
| Intracerebral+  Subdural | 4 (7.4%) | 1 (8.3%) | 3 (7.1%) |  |  |  |
| Intracerebral+  Subarachnoid | 3 (5.6%) | 0 (0.0%) | 3 (7.1%) |  |  |  |
| Total | 54 (100%) | 12 (100%) | 42 (100.0%) |  |  |  |

Type of ICH was not available in 16 cases (22.9%). Among 16 cases without ICH classification, 8 patients died at home, autopsy was performed and ICH was documented in the death certificate, 4 patients died at remote hospitals, and 4 had ICH admission at remote hospitals. Investigators made contact indexed hospital and confirmed that patients had ICH.

**Supplementary Table 2.** Univariable and multivariable analysis for predictors of intracranial hemorrhage in patients with oral anticoagulants

| **Variables** | **Univariate** | | **Multivariate** | |
| --- | --- | --- | --- | --- |
|  | **HR (95% CI)** | **p-value** | **HR (95% CI)** | **p-value** |
| Age (years) | 1.05 (1.02-1.07) | ***0.001**** | 1.05 (1.03-1.08) | ***<0.001**** |
| Female gender | 0.63 (0.37-1.06) | 0.081 | 0.45 (0.27-0.77) | ***0.004**** |
| Atrial fibrillation |  |  |  |  |
| - Paroxysmal | Reference | 0.487 |  |  |
| - Persistent | 1.24 (0.59-2.63) | 0.568 |  |  |
| - Permanent | 1.43 (0.80-2.58) | 0.232 |  |  |
| Symptomatic AF | 0.78 (0.45-1.34) | 0.366 |  |  |
| History of heart failure | 0.73 (0.41-1.34) | 0.326 |  |  |
| History of coronary artery disease | 0.75 (0.36-1.57) | 0.444 |  |  |
| Cardiac implantable electronic device | 0.86 (0.37-2.00) | 0.734 |  |  |
| History of ischemic stroke/TIA | 0.90 (0.48-1.69) | 0.749 |  |  |
| Hypertension | 1.23 (0.69-2.19) | 0.491 |  |  |
| Diabetes mellitus | 1.31 (0.78-2.21) | 0.309 |  |  |
| Smoking | 0.45 (0.19-1.03) | 0.060 | 0.33 (0.14-0.77) | ***0.010**** |
| Dyslipidemia | 1.51 (0.89-2.57) | 0.125 |  |  |
| Renal replacement therapy | 6.96 (2.18-22.19) | ***0.001**** | 7.89 (2.46-25.27) | ***0.001**** |
| Dementia | 1.71 (0.24-12.33) | 0.594 |  |  |
| CKD | 2.13 (1.24-3.64) | ***0.006**** |  |  |
| History of bleeding | 0.73 (0.29-1.82) | 0.496 |  |  |
| Antiplatelet | 1.04 (0.50-2.18) | 0.919 |  |  |

HR = hazard ratio, CI = confidence interval, AF = atrial fibrillation, TIA = transient ischemic attack, CKD = chronic kidney disease, DOAC = direct oral anticoagulants

**Supplementary Figure 1.** Flow diagram of study population

**
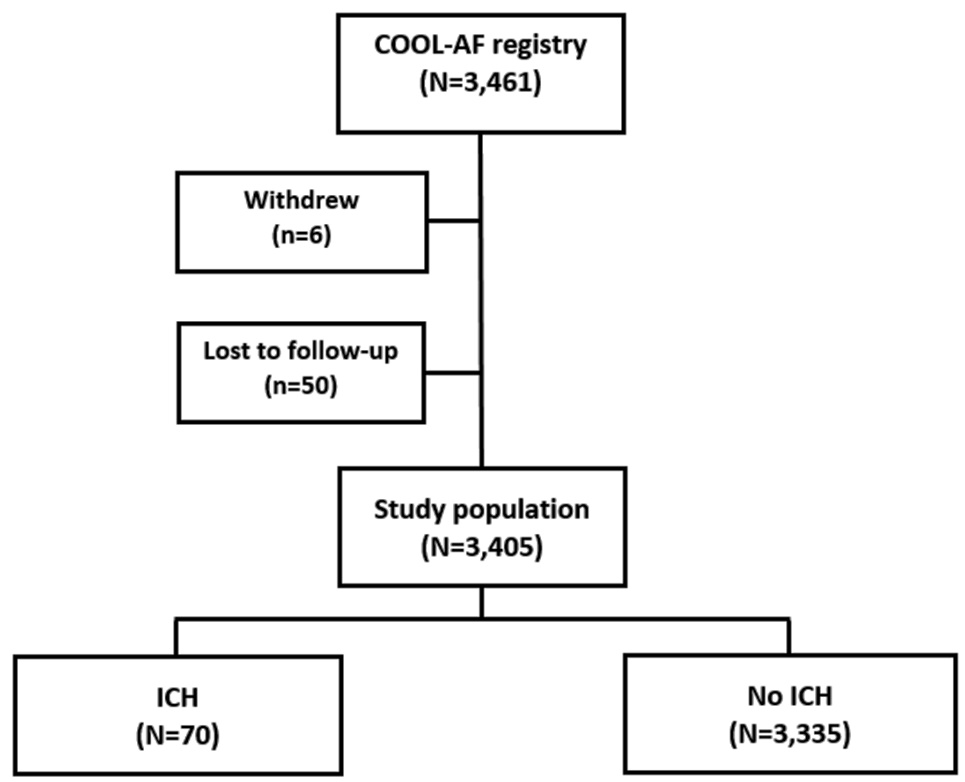
**

**Supplementary Figure 2.** Hazard graph of 5 risk groups (1 = very low risk, 2 = low risk, 3 = intermediate risk, 4 = high risk, and 5 = very high risk) for the prediction of intracranial hemorrhage. A. All patients B. Patients with oral anticoagulants (OAC)

**
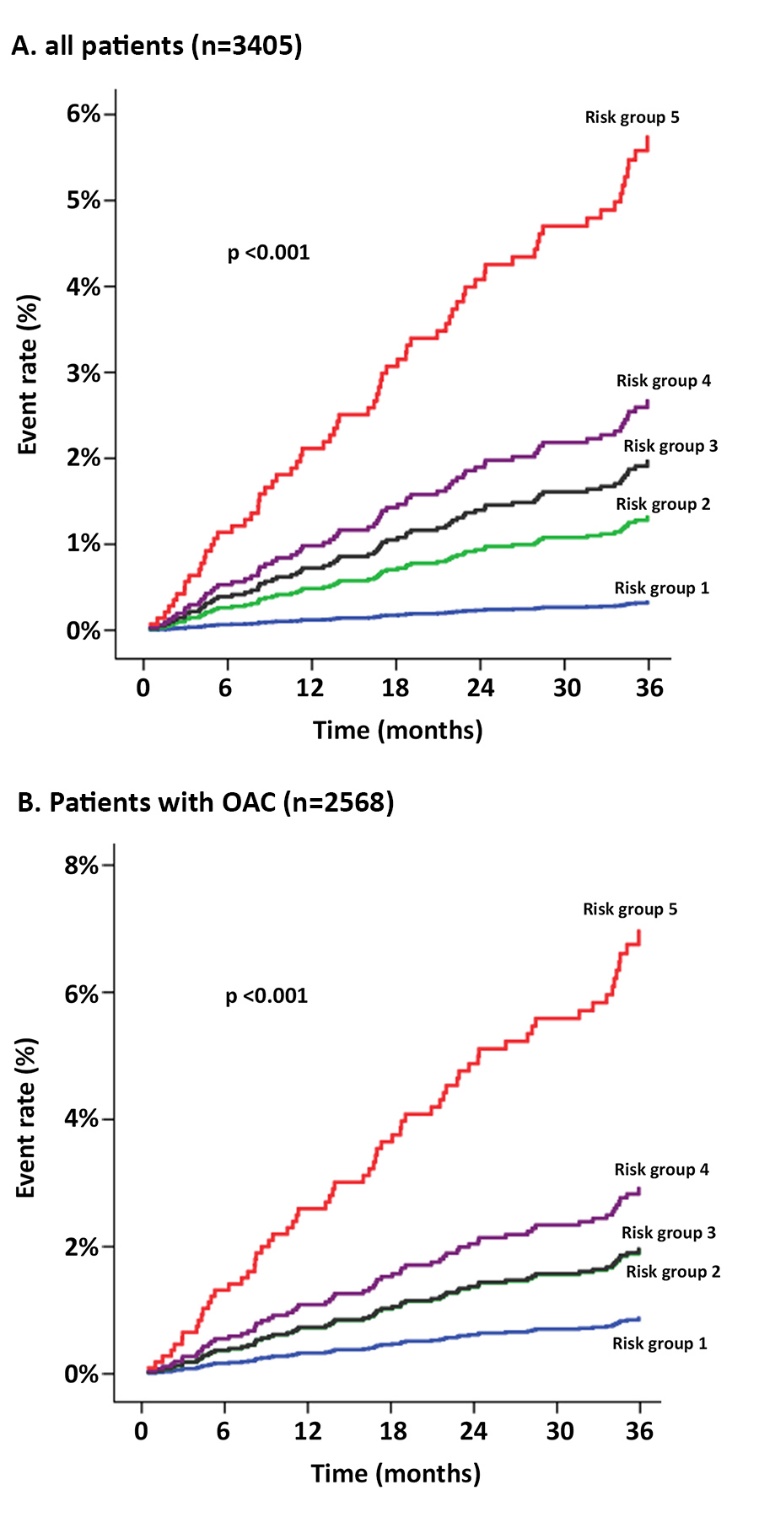
**

**Supplementary Figure 3.** Comparisons of the incidence rate of intracranial hemorrhage (ICH) between oral anticoagulants (OAC) and no OAC (left), warfarin and direct oral anticoagulants (DOACs) (middle) and warfarin with time in therapeutic range (TTR) < and ≥65% (right).


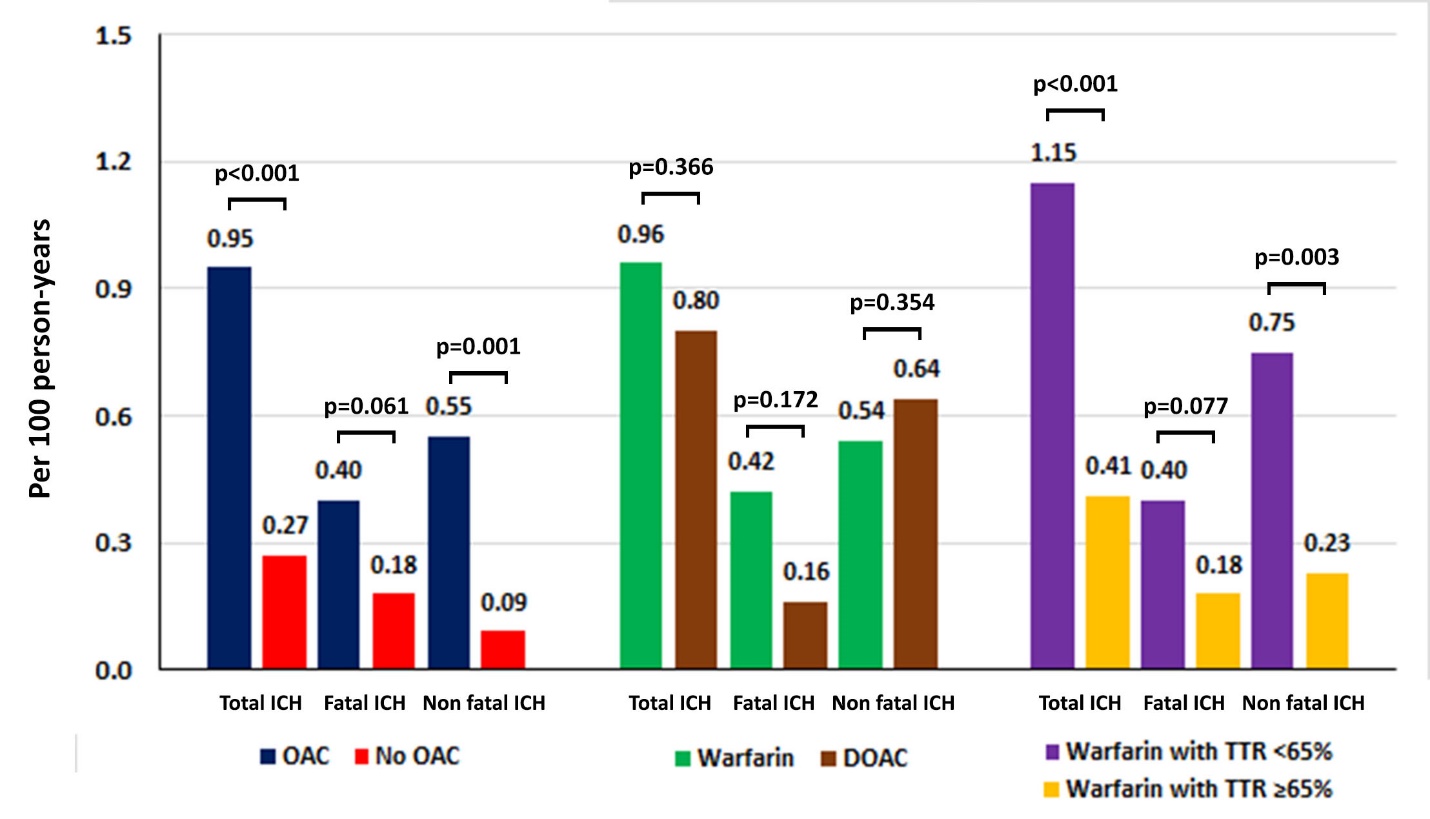

Supplement: Supplementary file 1 — Supporting information. [file CLC-47-e70040-s001.docx]
